# Gut Morphological Structure-Microbial Characteristics in *Elaphodus cephalophus*: A Case Report

**DOI:** 10.3390/ani15243651

**Published:** 2025-12-18

**Authors:** Siying Chen, Hao Dai, Zhiyu Feng, Haiyang Zhu, Jiahua Zhang, Tingting Fang, Shibin Yuan, Bangyuan Wu

**Affiliations:** 1 Key Laboratory of Southwest China Wildlife Resources Conservation, Ministry of Education, Nanchong 637000, China; 15328851098@163.com (S.C.); a2078590260@163.com (H.D.); smkxxyszfzb@163.com (Z.F.); 17879350898@163.com (H.Z.); zjhhua@yeah.net (J.Z.); fangtingting_cwnu@163.com (T.F.); 2 College of Life Science, China West Normal University, Nanchong 637000, China; 3 Nanchong Key Laboratory of Wildlife Nutritional Ecology and Disease Prevention and Control, Nanchong 637000, China

**Keywords:** structure of the gastrointestinal tract, gut microbiota, 16S rRNA, ITS fungal sequencing, *Elaphodus cephalophus*

## Abstract

The ruminant gastrointestinal tract hosts a complex microbial ecosystem vital for nutrient absorption, with each segment displaying distinct morphological and microbial compositional features compared to monogastric animals. While most studies rely on fecal samples, these fail to capture region-specific variations, limiting insight into microbe–physiology adaptations. In this study, we investigated *Elaphodus cephalophus* by measuring circular, longitudinal, and mucosal layer thickness across intestinal segments and by profiling bacterial and fungal communities via 16S and its rRNA sequencing. The results found that the stomach had the thickest circular (484.2 μm) and longitudinal (385.2 μm) muscle layers among all gastrointestinal segments. The thickness of the circular and longitudinal muscle layers in the stomach and duodenum showed a highly consistent variation trend (r > 0.74). Bacterial diversity was highest in the stomach and lowest in the ileum; cecal and rectal communities were similar but distinct from those in the duodenum, ileum, and stomach. Firmicutes and Bacteroidota dominated the bacterial phyla. Fungal abundance and diversity peaked in the cecum and were lowest in the stomach; Ascomycota was overall dominant, whereas Basidiomycota was most abundant in the duodenum. This study provides baseline descriptive data on the gastrointestinal muscle layer morphology and gut microbiota of *Elaphodus cephalophus*, establishing a basis for further study.

## Simple Summary

Most studies on herbivore gut microbiota rely solely on fecal samples, failing to capture the unique microbial communities across different segments of the digestive system. In this study, we examined the entire gastrointestinal tract of a wild tufted deer (*Elaphodus cephalophus*), an endangered small ruminant. We measured the thickness of the gut circular and longitudinal muscle layers and profiled bacterial and fungal communities in each segment using 16S rRNA and ITS sequencing. We found that the stomach had the thickest muscle layers among all gastrointestinal regions. Additionally, the types and abundances of bacteria and fungi varied significantly across different gut segments: Firmicutes and Bacteroidota dominated the bacterial community, while Ascomycota was the most abundant fungal phylum overall. These findings provide baseline descriptive data on the gastrointestinal morphology (focusing on muscle layers) and gut microbiota of this endangered species, supplementing existing knowledge that is mostly based on fecal samples.

## 1. Introduction

The animal gastrointestinal tract comprises intricate microbial communities that are crucial for host nutrient absorption, immune regulation, and metabolic functions. As herbivores reliant on microbial fermentation to break down fibrous substances, the composition and role of ruminants’ intestinal flora significantly influence the host’s physiological health and productivity [1]. Ruminants possess a highly specialized gastrointestinal tract, an evolutionary adaptation to a diet rich in fiber and plant material. Fibrous substances are primarily degraded through compartmentalized fermentation within the complex gastric system of the hairy crested deer (*Elaphodus cephalophus*), which includes the rumen, reticulum, omasum, and abomasum. The rumen serves as the central fermentation chamber, providing an anaerobic environment and sufficient retention time for microbial breakdown of cellulose and hemicellulose. In contrast, the small intestine—comprising the duodenum, jejunum, and ileum—is responsible for nutrient absorption, while the cecum and large intestine contribute to secondary fermentation and water reabsorption [2,3]. Structural and functional variations across these regions create distinct physicochemical environments (such as pH gradients, oxygen levels, and retention times), resulting in a unique microbial biogeographic distribution within the gastrointestinal tract [4,5].

Numerous studies have shown significant segmental differences in microbial composition, function, and diversity in ruminants, with their gut microbial diversity and abundance generally higher than in monogastric animals due to reliance on microbial fermentation for energy [6,7]. Investigating microbial composition, spatial distribution, and biogeographic features across gastrointestinal segments is essential to describe the characteristics of gut microbiota in ruminants [8]. While fecal samples are non-invasive, they only reflect terminal gut microbiota and fail to capture heterogeneity in upstream segments (e.g., stomach, small intestine, cecum)—a critical limitation for ruminants with multicompartmental stomachs and segmented intestinal functions, as key fermentation processes and microbial functions at sites like the rumen and cecum may be overlooked [9].

*Elaphodus cephalophus*, a Chinese endemic secondary protected small ruminant inhabiting subtropical scrub, feeds primarily on high-fiber, low-nutrient foods (wild fruits, seeds, fungi) and plays an ecological role in seed dispersal [10]. Its complex gastric structure, intestinal length ratio, and well-developed cecum are adapted for high-fiber digestion and secondary fermentation [11,12], with intestinal microorganisms driving high-fiber degradation and segment-specific microbiota linked to digestive function adaptation [13]. Studying its full gastrointestinal microbial communities is valuable for revealing gut structure-microbe co-adaptation in wild ruminants: the stomach’s thick muscular layer creates a homogeneous bacterial environment via peristalsis, while the jejunum’s thick mucus layer facilitates microbe-nutrient interactions [14]. Additionally, segment-specific bacteria (including *Escherichia-Shigella*) and fungi are closely tied to nutrient absorption and immune regulation [15,16], with Ascomycota and Basidiomycota synergistically degrading lignocellulose to aid plant material utilization [17]. A comprehensive understanding of its microbial biogeography may also inform captive feed development (e.g., fiber-degrading flora transplantation) and disease control [18,19]. However, research integrating macroscopic intestinal structure, whole-tract microbial composition, and fungal communities remains limited—addressing this gap is the focus of the present study.

In this study, we analyzed the bacterial and fungal communities throughout the entire gastrointestinal tract of wild *Elaphodus cephalophus*, including the four chambers of the stomach, the three segments of the small intestine (Duodenum, Jejunum, Ileum), and the large intestine (Cecum, Colon, Rectum), using high-throughput sequencing. This analysis was complemented by histomorphometric observations of the gastrointestinal tract. We integrated these approaches at both the microbial distribution level and the macroscopic organizational and structural adaptation levels. The aims of this study were to characterize the spatial distribution patterns of microorganisms, clarify compositional differences between bacteria and fungi at the phylum and genus levels, identify core dominant taxa, and elucidate the adaptive relationships between the functions of zone-specific microbiota and the physiological structure and function of the host gastrointestinal tract.

## 2. Materials and Methods

### 2.1. Sample Collection

During a field survey in a nature reserve, the carcass of an adult male *Elaphodus cephalophus* (approx. 5 years old) was found, which had died from accidental drowning. The carcass was still palpably warm at discovery, suggesting a relatively short post-mortem interval (PMI); while this implies that the gut microbiota may not have undergone substantial succession or putrefaction, this inference lacks direct experimental validation. The individual was promptly transferred to a preservation station located less than 50 m from the site of death for autopsy under sterile conditions. Contents from distinct segments of the gastrointestinal tract (GIT)—encompassing the rumen, reticulum, omasum, abomasum, duodenum, jejunum, ileum, cecum, colon, and rectum—were systematically harvested. Following precise anatomical identification of each segment, content samples were aseptically collected into sterile tubes and labeled sequentially, resulting in a total of 21 samples (specifically, two samples each from the rumen, reticulum, omasum, abomasum, jejunum, ileum, cecum, colon, and rectum and three samples from the duodenum). Parallel tissue samples were immersion-fixed in 4% paraformaldehyde for subsequent histological processing. All content samples were transported to the laboratory on dry ice and stored at −80 °C for DNA extraction.

It should be noted that this study has inherent limitations associated with the sampling context: (1) Given the individual’s drowning cause of death, there exists a potential risk of water-borne microbial contamination or aspiration of environmental microbes into the gastrointestinal tract during the drowning process, which may have altered the in vivo microbial profiles; (2) Even with a short PMI, early post-mortem shifts in the gut microbial community cannot be completely excluded; (3) All observational findings of this study only represent the gastrointestinal morphology and putative microbial patterns of this individual drowned *Elaphodus cephalophus*, and only serve as key controls and references for subsequent similar studies.

### 2.2. Observation of Gastrointestinal Tissue Structure

Fixed GIT tissues underwent a standard dehydration process through a graded ethanol series (50%, 75%, 85%, 95%, and 100%), were cleared in xylene, and embedded in paraffin. Sections were prepared from the embedded blocks using a microtome. Following sectioning, tissues were stained with hematoxylin and eosin (H.E.), coverslipped, and examined under a microscope. This allowed for detailed morphological observation and measurement of the thicknesses of the circular muscle layer, longitudinal muscle layer, and mucosal layer for subsequent analysis.

### 2.3. Gastrointestinal Microbial DNA Extraction

Total genomic DNA from the gastrointestinal microbiota was isolated employing the QIAamp PowerFecal Pro DNA Kit (Cat. No. 51804, Qiagen, Hilden, Germany). The integrity of the extracted DNA was verified via 1% agarose gel electrophoresis, while concentration and purity were determined using a NanoDrop 2000 spectrophotometer (Thermo Fisher Scientific, Waltham, MA, USA).

### 2.4. Bacterial PCR Amplification and Illumina MiSeq Sequencing

The hypervariable V3–V4 regions of the bacterial 16S rRNA gene were amplified using the primer pair 338F (5′-ACTCCTACGGGGAGGCAGCAG-3′) and 806R (5′-GGACTACHVGGGTWTCTAAT-3′). Amplifications were carried out in an ABI GeneAmp^®^ 9700 PCR Thermal Cycler (ABI Corporation, Los Angeles, CA, USA) with the following program: 95 °C for 3 min; 29 cycles of 95 °C for 30 s, 55 °C for 30 s, and 72 °C for 30 s; a final extension at 72 °C for 10 min; and a 4 °C hold. Each 20 µL PCR reaction comprised: 4 µL of 5× TransStart FastPfu buffer, 2 µL of 2.5 mM dNTPs, 0.8 µL of each primer (5 µM), 0.4 µL of TransStart FastPfu DNA Polymerase, 10 ng of template DNA, and ddH_2_O to volume. Triplicate PCRs were performed for each sample. The resulting amplicons were pooled, purified, and quantified. Equimolar amounts of purified PCR products from all samples were combined and sent to Meijer Biopharmaceuticals (Shanghai, China) for paired-end sequencing (2 × 300 bp) on an Illumina MiSeq PE300 platform (San Diego, CA, USA).

### 2.5. Fungal PCR Amplification, Illumina MiSeq Sequencing

The fungal ITS1 region was amplified using primers ITS1F (5′-CTTGGTCATTTAGAGGAAGTAA-3′) and ITS2R (5′-GCTGCGTTCTTCATCGATGC-3′). The thermal cycling conditions on the ABI GeneAmp^®^ 9700 PCR thermocycler were: 95 °C for 3 min; 35 cycles of 95 °C for 30 s, 55 °C for 30 s, and 72 °C for 30 s; a final extension at 72 °C for 10 min; and a 4 °C hold. The 20 µL reaction mixture composition was identical to that described for bacterial amplification. All reactions were performed in triplicate. Post-amplification, products were purified, quantified, and pooled in equimolar ratios. The final library was sequenced on the Illumina MiSeq PE300 platform by Meijer Biopharmaceuticals Co., Ltd. (Shanghai, China).

### 2.6. Data Analysis of Sequencing Results

Paired-end reads from sequencing were initially merged based on overlap, followed by demultiplexing and quality filtering using default parameters. Subsequent analysis included Operational Taxonomic Unit (OTU) clustering, alternatively Amplicon Sequence Variant (ASV) denoising, and taxonomic classification. Diversity indices (α-diversity) were calculated from the OTU/ASV tables after rarefaction. β-diversity was assessed based on Bray–Curtis distances, and Principal Coordinate Analysis (PCoA) was performed to visualize community dissimilarities among gastrointestinal segments. All statistical analyses and visualizations were conducted in R (v3.6.0) utilizing the vegan (v3.1.1) and ggplot2 (v2.5-6) packages. Taxonomic assignment was performed against the SILVA database (v138) for 16S data and the UNITE database for fungal data using a Naïve Bayesian classifier with K-mer-based alignment (k = 8) [20].

### 2.7. Analysis of Slice Result Data

Digital images of tissue sections (Four replicate tissue sections were prepared for each intestinal segment) were acquired under consistent bright-field illumination, ensuring that the entire cross-section of the tissue was captured in the field of view. Five non-overlapping, intact villus regions were captured per slide, with each gastrointestinal tissue segment is equipped with a corresponding micron-scale scale bar. The thicknesses of the muscular and mucosal layers were measured in micrometers (μm) using ImageJ software (v1.54p, NIH, Bethesda, MD, USA). During measurement, artifacts and oblique sections were carefully avoided, and the crypt basement membrane was used as a reference standard. Data were compiled in Excel to calculate the mean values and standard deviations (Mean ± SD) for each gastrointestinal (GIT) segment.

## 3. Results

### 3.1. Gastrointestinal Structural Features and Their Correlation

#### 3.1.1. Gastrointestinal Histological Structure of *Elaphodus cephalophus*

**Figure 1:**
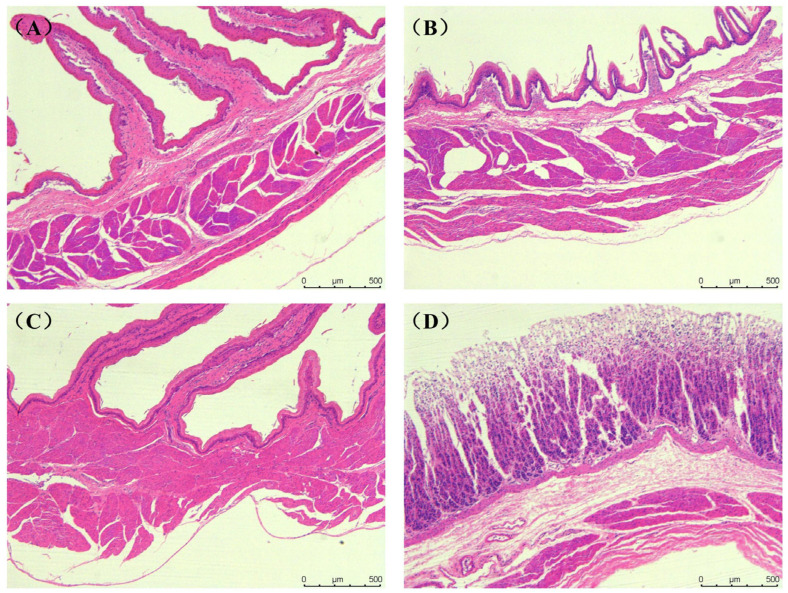
Histological structure of the gastric tissue of the *Elaphodus cephalophus*: (**A**) Abomasum, (**B**) Reticulum, (**C**) Omasum, and (**D**) Rumen. (50×, H.E.).

**Figure 2:**
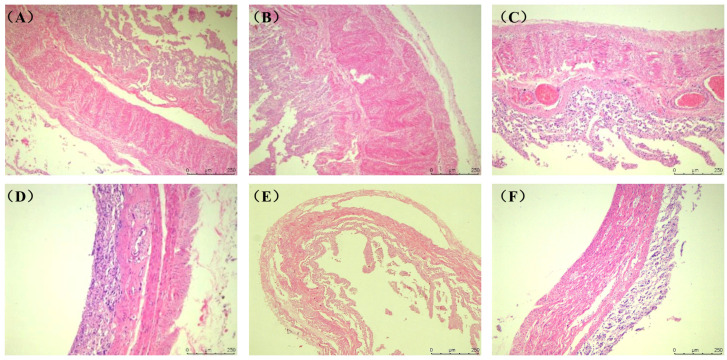
Histological structure of the digestive tract of the *Elaphodus cephalophus* (**A**) Duodenum. (**B**) Jejunum. (**C**) Ileum. (**D**) Cecum. (**E**) Colon. (**F**) Rectum. (100×, H.E.). (Note: Autolysis of the small intestinal mucosa (**A**–**C**) is observed, leading to unreliable mucosal thickness measurements).

The tissue structure of the rumen, reticulum, omasum, abomasum and duodenum of *Elaphodus cephalophus* mainly consisted of the mucous membrane layer and the muscle layer (the circular and longitudinal muscle layers) (Figure 1A–D). The tissue structure of the jejunum, ileum, cecum, colon and rectum mainly consisted of the mucous membrane layer and the muscle layer (the circular and longitudinal muscle layers), as shown in Figure 2A–F.

#### 3.1.2. The Thickness of the Circular and Longitudinal Muscle Layers of the Gastrointestinal Tract in the *Elaphodus cephalophus*

**Figure 3:**
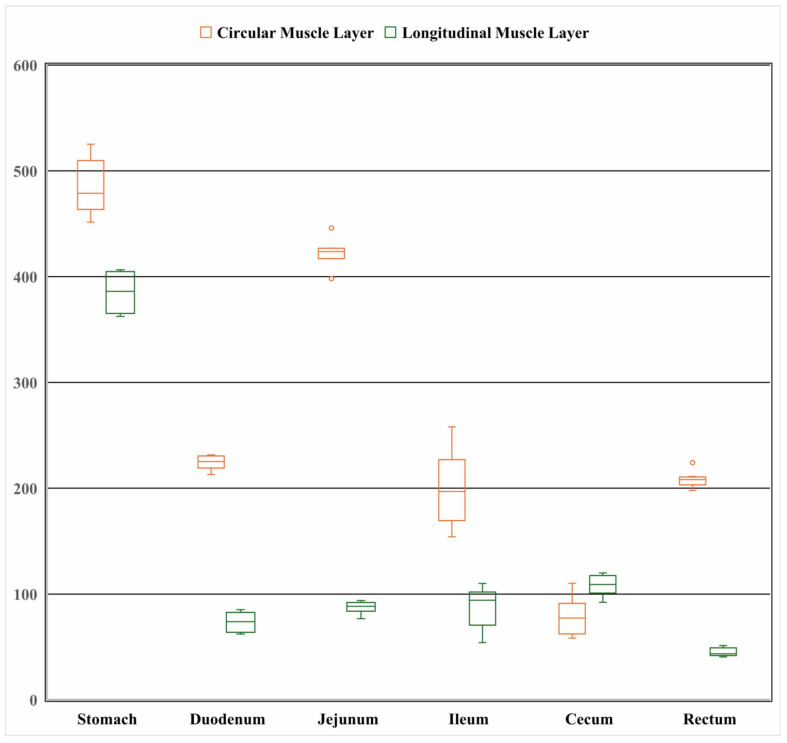
Thickness of the circular and longitudinal muscle layers of the gastrointestinal tract of the *Elaphodus cephalophus* (μm).

As shown in Figure 3, the gastric circular muscle was the thickest (484.29 ± 25.05 μm), approximately 6.1 times thicker than that of the cecum (79.04 ± 17.00 μm). Among the small intestine segments, the jejunal circular muscle was the thickest (422.40 ± 14.02 μm), while the circular muscle of the large intestine was relatively thin (79.04 ± 17.00 μm). In terms of the longitudinal muscle layer, the stomach had the thickest longitudinal muscle (385.08 ± 16.69 μm), approximately 8.6 times thicker than that of the rectum (44.90 ± 3.78 μm). The longitudinal muscle thickness of the small intestine segments was similar (jejunum = ileum > duodenum), and the cecum had a relatively thicker longitudinal muscle layer. Overall, the thickest structure among the measured muscle layers was the gastric circular muscle layer (484.3 μm), while the thinnest was the rectal longitudinal muscle layer (44.8 μm). (Note: Autolysis of the small intestinal mucosa (Figure 2A–C) was observed, rendering mucosal thickness measurements unreliable; thus, mucosal layer data are not presented herein.)

#### 3.1.3. Correlation Characteristics of the Circular and Longitudinal Muscle Layers in the Gastrointestinal Tract of *Elaphodus cephalophus*

In *Elaphodus cephalophus*, the muscular layers (circular and longitudinal) of the stomach and duodenum exhibited a strong positive correlation (r > 0.74, *p* < 0.05). The longitudinal muscle layers of the rectum showed high synergistic correlation (r > 0.73) (Table 1). The thickness of the gastric circular muscle layer was greater than that of the duodenal circular muscle layer and the small intestinal circular muscle layer. The correlation coefficient between the circular and longitudinal muscle layers in the posterior segment of the small intestine was r = 0.556. Correlation characteristics of other gastrointestinal segments are provided in the Supplementary Materials (Supplementary Tables S1 and S2).

### 3.2. Bacterial Community Composition and Diversity

**Figure 4:**
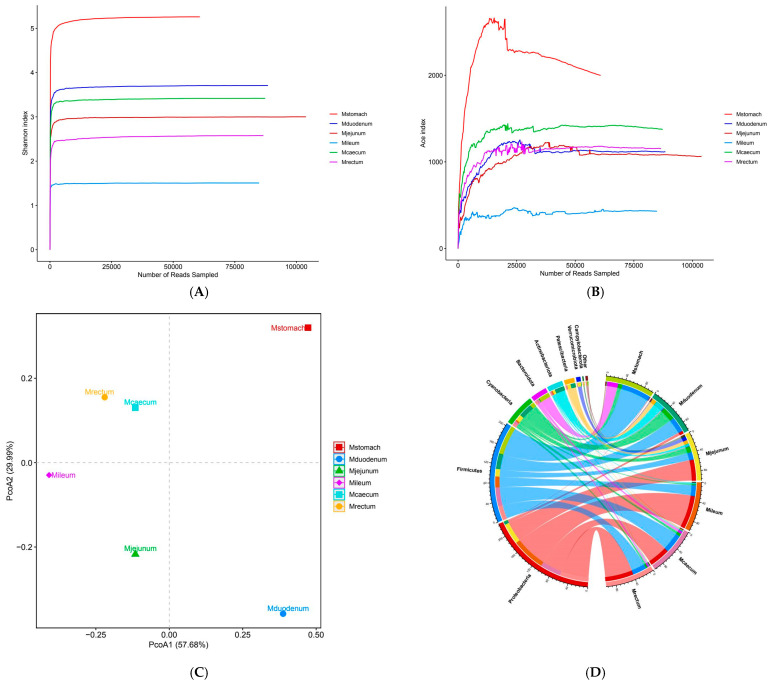

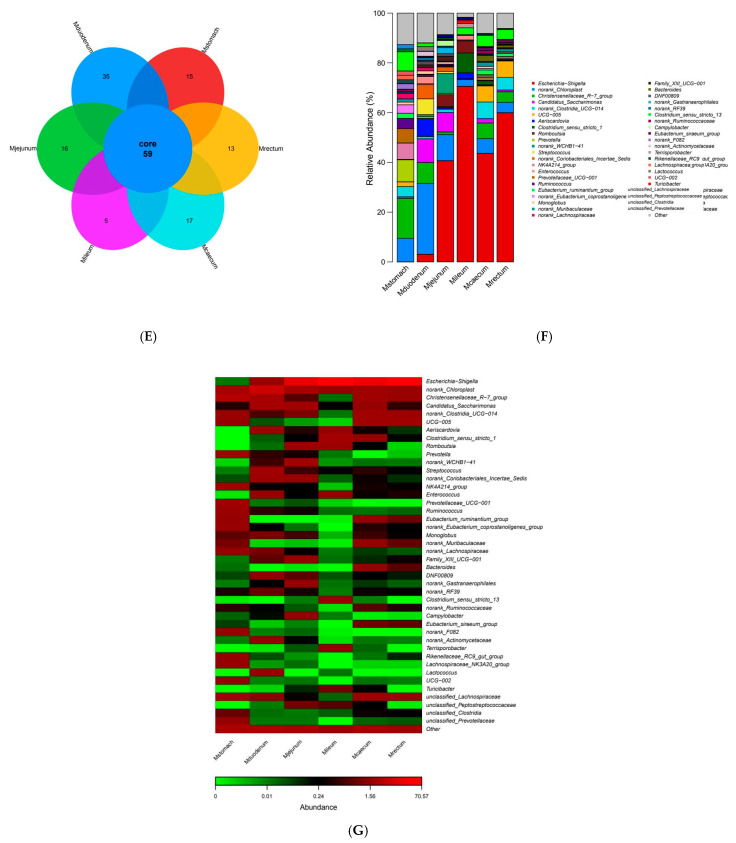
Gastrointestinal bacterial diversity. (**A**) Alpha index dilution curve. (**B**) Shannon index dilution curve. (**C**) PCoA: principal coordinate analysis. (**D**) Collinearity relationship diagram. (**E**) Venn diagram of species distribution among samples. (**F**) Relative abundance bar chart of dominant species. (**G**) Abundance Heatmap.

The dilution curves for each sample site tended to flatten out, according to 16S sequencing results, suggesting that the sequencing depth captured the majority of species and that the sequencing data for each sample were sufficiently large (Figure 4A). The stomach had the greatest overall bacterial diversity, followed by the duodenum, and the ileum had the lowest (Figure 4B). The bacterial compositions of the duodenum, ileum, and stomach varied considerably, although the cecum and rectum were rather similar, according to the PCoA plot based on Bray–Curtis distance (Figure 4C). The six phyla that were most prevalent at the phylum level were Firmicutes, Proteobacteria, Cyanobacteria, Bacteroidetes, Actinobacteria, and Proteobacteria, in order of decreasing abundance. Out of these, the phyla Firmicutes and Proteobacteria showed a notable advantage over the others (Figure 4D). At the genus level, *Escherichia-Shigella*, norank_Chloroplast, Christensenellaceae_R-7_group, Candidatus_Saccharimonas, norank_Clostridia_UCG-014, UCG-005, and *Aeriscardovia* were the most prevalent genera in the gastrointestinal tract (Figure 4F). Bacterial abundance varies across different gastrointestinal regions, with *Escherichia-Shigella* being most abundant in the jejunum, ileum, cecum, and rectum, reaching the highest relative abundance in the ileum (70.57%), followed by the rectum (59.98%), and the lowest in the duodenum (3.09%). It is not present in the stomach (Figure 4F). Christensenellaceae_R-7_group is the most abundant genus in the stomach, accounting for only 16.12%. The species richness in the stomach is significantly higher than in other intestinal segments. In the duodenum, except for norank_Chloroplast (28.43%), the remaining major genera each account for approximately 10%, with no dominant genus. Bacterial species richness is second only to the stomach and higher than in other intestinal segments (Figure 4F). There were distinct genera for every intestinal segment. Candidatus_Saccharimonas (9.52%), *Aeriscardovia* (7.1%), and norank_Actinomycetaceae (1.92%) were unique genera in the duodenum (Supplementary Figure S1A), while Christensenellaceae_R-7_group, *Prevotella* (8.88%), unclassified_Lachnospiraceae (7.74%) were unique bacterial genera in the stomach (Supplementary Figure S1B), UCG-005 (6.57%), *Bacteroides* (1.03%) were unique bacterial genera in the rectum (Supplementary Figure S1C), norank_WCHB1-41 (8.04%), *Romboutsia* (4.86%), and norank_Gastranaerophilales (2.43%) were unique bacterial genera in the jejunum (Supplementary Figure S1D), *Clostridium_sensu_stricto_1* (7.98%), *Romboutsia* (4.89%), *Terrisporobacter* (1.77%) were unique genera of the ileum (Supplementary Figure S1E), norank_Clostridia_UCG-014 (6.7%), UCG-005 (6.45%), norank_Ruminococcaceae (1.03%) were unique genera of the cecum (Supplementary Figure S1F).

Each gastrointestinal region’s common and distinct bacterial genera were depicted using Venn diagrams. The findings revealed that 59 bacterial genera, or 36.87% of the total, were shared by the five sample groups. Of these, the duodenum had the most species (35), and the cecum the second-most (17), the ileum the fewest (5) (Figure 4E). The bacterial species found in the various gastrointestinal regions varied significantly (Figure 4G).

### 3.3. Fungal Community Composition and Diversity

**Figure 5:**
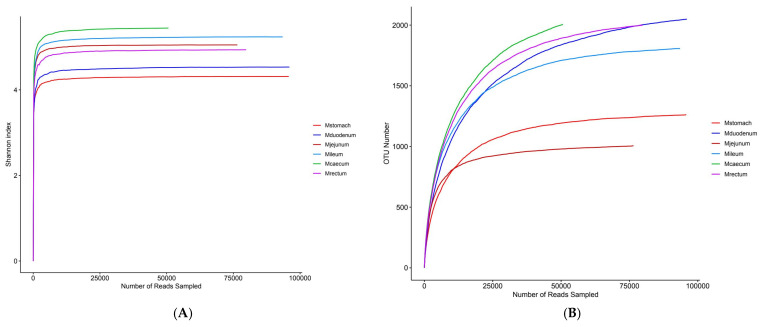

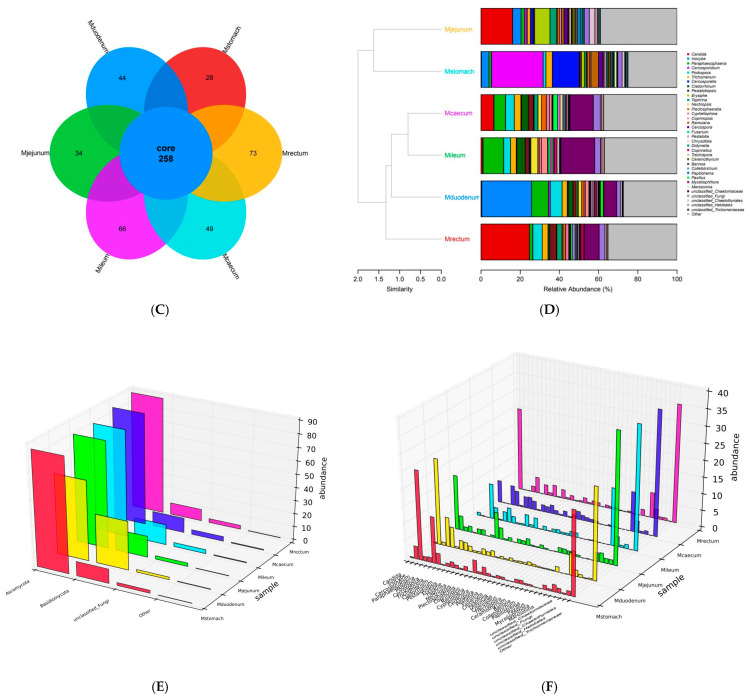

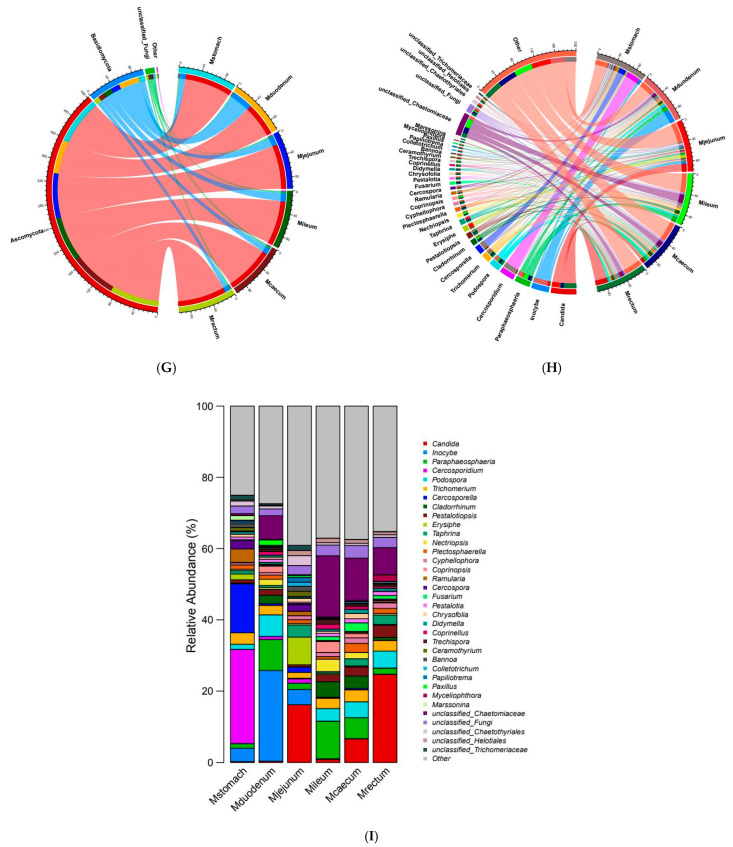
Gastrointestinal fungal diversity (**A**) Shannon diversity index dilution curve. (**B**) Shannon’s evenness dilution curve. (**C**) Gastrointestinal genus-level species Venn diagram. (**D**) Similarity of gastric and intestinal fungal genus-level species. (**E**) Gastrointestinal fungal phylum-level abundance 3D bar chart. (**F**) 3D bar chart of fungal genus-level abundance in the gastrointestinal tract. (**G**) Correlation diagram of fungal phylum-level abundance in the gastrointestinal tract. (**H**) Correlation diagram of fungal genus-level abundance in the gastrointestinal tract. (**I**) Relative abundance diagram of fungal genus-level abundance in the gastrointestinal tract.

The curves for the stomach, duodenum, jejunum, ileum, caecum, and rectum all have a tendency to flatten out, according to its sequencing results, suggesting that the sequencing depth covers the majority of species and the sequencing data volume is adequate (Figure 5A). The caecum had the highest levels of fungal richness and evenness, followed by the ileum, jejunum, rectum, and duodenum. The stomach had the lowest levels. This reflects the different dominance of particular fungal species in each intestinal segment and shows notable changes in fungal species richness and evenness between intestinal segments (Figure 5B).

In the analysis of gut microbiota composition, at the phylum level, there were primarily three phyla, ranked by abundance as follows: Ascomycota, Basidiomycota, and unclassified fungi (Figure 5G). Ascomycota had the highest abundance and is the dominant phylum in the gut microbiota (Supplementary Figure S2G), with the highest abundance in the rectum (88.07%). The duodenum (60.72%) had relatively lower abundance, but Basidiomycota (basidiomycetes) had the highest abundance in the duodenum (37.05%) (Figure 5E).

At the genus level, Chaetomiaceae (a family-level taxon) and *Paraphaeosphaeria* were the most dominant genera, while the species *Candida albicans* also exhibited high abundance. *Candida albicans* and Chaetomiaceae belong to the Ascomycota phylum, and *Paraphaeosphaeria* also falls under the Ascomycota phylum (Figure 5H). Unclassified-Chaetomiaceae is primarily distributed in the duodenum, jejunum, ileum, and rectum. *Paraphaeosphaeria* is primarily distributed in the duodenum, ileum, and cecum (Supplementary Figure S2H). *Candida* is the dominant genus in the jejunum, cecum, and rectum, while *Inocybe* (25.41%) is the dominant genus in the duodenum and is present in small quantities in the stomach (3.77%) and jejunum (4.28%) (Figure 5F). *Cercosporidium* (26.4%) and *Cercosporella* (13.76%) were the dominant genera in the stomach, *Erysiphe* (7.77%) is more prevalent in the jejunum, and is only present in the stomach (1.62%) (Figure 5I).

A total of 258 OTUs were shared by the stomach and other intestinal segments. Every segment had distinct fungal genera, with the rectum having the most unique genera (73), which make up 22% of the rectum’s total OTUs. The ileum came next, with 66 distinct OTUs, or 20% of the total (Figure 5C). The species composition of the stomach and different intestine segments, as well as the intestinal segments themselves, varied significantly. However, the cecum and ileum, as well as the stomach and jejunum, exhibited relatively high species similarity (Figure 5D).

At the same time, different unique genera were found in the stomach and various intestinal segments of the *Elaphodus cephalophus*, except for the ileum (Supplementary Figure S2E). *Marssonina* (1.27%) is a unique genus in the stomach (Supplementary Figure S2A), and *Paxillus* (1.55%) is a unique genus in the duodenum (Supplementary Figure S2B). *Colletotrichum* (1.12%) and *Papiliotrema* (1.39%) (1.39%) were unique to the jejunum (Supplementary Figure S2C), *Nectriopsis* (1.76%) and *Didymella* (1.11%) were unique to the cecum (Supplementary Figure S2D), and *Myceliophthora* (1.88%) is unique to the rectum (Supplementary Figure S2F).

## 4. Discussion

This case report describes the compositional and distributional differences in the gut microbiota in *Elaphodus cephalophus* alongside the morphological characteristics of the gastrointestinal muscle layers, and these observations may provide clues for understanding potential links that could be validated in future studies. The physiological functions of the circular and longitudinal muscle layers might be potentially related to gut microbial activity, and this raises the possibility that microorganisms in different intestinal segments could play roles in regulating intestinal and systemic health—an area worthy of further investigation. Histological examination of gastrointestinal sections from *Elaphodus cephalophus* revealed that both the circular and longitudinal muscle layers in the stomach are significantly thicker than those in other intestinal regions. The circular muscle layer regulates luminal diameter, while contraction of the longitudinal muscle shortens intestinal length; their coordinated action may modulate chyme propulsion [21], which could potentially contribute to mechanical digestion and the maintenance of a relatively stable substrate environment—hypotheses that require further testing [22]. The jejunal mucosa has been reported to serve as a critical site for nutrient absorption and an essential component of the intestinal immune system [23], and the unique genera identified in the jejunum (exclusively anaerobic Firmicutes: norank_WCHB1-41: 8.04%; *Romboutsia*: 4.86%; norank_Gastranaerophilales: 2.43%) might be associated with these functions. Previous studies have shown that short-chain fatty acids (SCFAs) produced by anaerobes can enhance mucosal immunity and barrier integrity by modulating intestinal permeability [24], and this mechanism could potentially align with the structural features of the jejunum, though this association needs to be verified in subsequent research. The well-developed muscle layers in the stomach and the specialized anaerobic microbiota in the jejunal region may collectively contribute to nutrient absorption efficiency and mucosal immune barrier function in *Elaphodus cephalophus*, providing a potential direction for future functional validation studies.

This study was conducted in Sichuan Ya’an Baoxing, with samples collected from an adult male *Elaphodus cephalophus* in autumn. As a typical herbivore, *Elaphodus cephalophus* is known to exhibit dietary variability corresponding to seasonal changes in environmental plant resources in the wild [25]. Such potential seasonal shifts in food availability might impose selective pressure on the structure and function of its gut microbiota over evolutionary time, which could favor the development of region-specific microbial communities that may be associated with the physiological differentiation along the gastrointestinal tract [26]—a hypothesis that could be explored through multi-seasonal studies in the future.

*Elaphodus cephalophus* relies on plant-based resources as its primary diet, and high-throughput sequencing revealed significant differences in bacterial composition at both phylum and genus levels, particularly among the duodenum, ileum, and stomach. Bacterial diversity was highest in the stomach and lowest in the ileum; the microbial communities of the cecum and rectum clustered closely, while those of the duodenum, ileum, and stomach were clearly distinct. As the primary site of fermentation initiation, the stomach is exposed to diverse plant substrates, and its high bacterial diversity might facilitate the breakdown of complex plant materials—a potential functional association that warrants further investigation. At the phylum level, Firmicutes and Bacteroidota dominated and constitute the core phyla in ruminants [27]. They have been reported to be involved in food digestion and absorption, energy metabolism, and pathogen defense [23], with synergistic activities that may enhance the digestion of both proteins and carbohydrates [28]. These phyla are integral to the gut microbiota of *Elaphodus cephalophus*, and their dominance might be associated with the host’s dietary habits, which could be explored in future studies on high-fiber diet adaptation. Firmicutes have been documented to efficiently degrade dietary fiber, and high-fiber intake may promote their enrichment [28], while Bacteroidota could regulate carbohydrate metabolism, help maintain microbial equilibrium, and enhance host immunity [23]—a role further evidenced by their dominance in the caprine rumen [29]. The stomach of *Elaphodus cephalophus* was notably dominated by Christensenellaceae_R-7_group, which is also a predominant genus in the stomach of sika deer [30], and this consistency might indicate a potential adaptation of gut microbiota to the natural environment. Its high relative abundance (16.12%) could be attributed to high efficiency in utilizing homogenized fiber substrates in the stomach, suggesting a potential key role in digesting fibrous foods that requires validation through functional experiments. Microbe-mediated immune regulation may have potential correlations, and microbial metabolites may in turn affect gastric motility, forming a bidirectional feedback loop through neuro-immune-endocrine interactions to regulate gastrointestinal motility, fluid transport and blood flow [31]. These potential mechanisms, if confirmed, could help explain how the gut microbiota facilitates the adaptation of *Elaphodus cephalophus* to its specific ecological environment, providing valuable hypotheses for future research.

*Escherichia-Shigella* is widely distributed in the small and large intestines of *Elaphodus cephalophus*, with the highest relative abundance in the ileum (70.57%), followed by the rectum (59.98%), and the lowest in the duodenum (3.09%). In contrast to the general trend in other ruminants, where the abundance of this taxon decreases with age [32], the unusually high colonization levels in the ileum and rectum of *Elaphodus cephalophus* might reflect a host-specific adaptation, suggesting that this microbial group could have evolved a mutually beneficial ecological function rather than a pathogenic relationship within this host—an assumption that requires further verification. The ileum is the major site of terminal absorption of glucose and amino acids [33], and this nutritional environment might be well-suited to the potential ability of *Escherichia-Shigella* to efficiently degrade substrates and produce short-chain fatty acids (SCFAs). The rectal environment, characterized by prolonged content retention, low oxygen levels, and abundant shed epithelial cells and mucus [14], could favor the growth of facultative anaerobes such as *Escherichia-Shigella*. In contrast, the physiological environment of the duodenum might impose strong constraints on this taxon: its dense vascular network [34] and well-developed villi [35] create the highest oxygen tension in the gastrointestinal tract [36], which could be unfavorable for the proliferation of facultative anaerobes. Additionally, the rapid transit of contents and sharply elevated pH (to neutralize gastric acid) in the duodenum might effectively inhibit the colonization of most incoming bacteria [37,38]. These conditions—high oxygen, high pH, and rapid peristalsis—might collectively contribute to the lowest abundance of *Escherichia-Shigella* in the duodenum of *Elaphodus cephalophus*, providing a potential explanatory framework for future studies to test. Although the duodenal microbiota lacks a dominant genus, it contains the highest number of unique taxa and has bacterial richness second only to the stomach. Key unique genera include *Candidatus Saccharimonas* (9.52%), *Aeriscardovia* (7.1%), and norank_Actinomycetaceae (1.92%). Saccharimonas is an obligate epibiotic parasite that attaches to actinobacterial hosts (such as *Aeriscardovia* and Actinomycetaceae) via type IV pili [39,40], which might imply its functional importance in the duodenum. This genus has been reported to synthesize various enzymes, amino acids, and vitamins; metabolize luminal compounds (e.g., lactate); and provide nutritive metabolites to the host [41,42,43,44], and these traits could explain its potential adaptation to nutrient-poor and rapid-transit environments like the duodenum. Reduced abundance of Saccharimonas has been associated with inflammatory bowel disease (IBD) [15], suggesting a potential role in maintaining microbial balance and local immunity through symbiotic interactions. These observations could offer valuable insights for future research on the functional roles of duodenal microbiota in *Elaphodus cephalophus*. Although considerable microbial overlap exists between the small and large intestines, both differ significantly from the gastric microbiota, which might indicate distinct functional potentials across gut segments that merit further exploration.

Fungal richness and evenness were highest in the cecum and lowest in the stomach. At the phylum level, the fungal community was predominantly composed of Ascomycota and Basidiomycota. Ascomycota was overall the most dominant phylum, reaching its highest abundance in the rectum (88.07%), while Basidiomycota was relatively enriched in the duodenum, where it exhibited its peak abundance (37.05%). Ascomycota and Basidiomycota are the most abundant fungi in topsoil [45]. Ascomycota has been reported to primarily degrade plant cell wall polysaccharides (such as cellulose, hemicellulose, and pectin), and its high abundance in the rectum (88.07%) might indicate a potential important role in hindgut fermentation. Basidiomycota is typically known for its activity in lignin degradation, and its higher abundance in segments such as the duodenum (37.05%) might be related to specific dietary components ingested by *Elaphodus cephalophus* or to the intestinal microenvironment [45]. These two phyla could collectively or sequentially participate in plant residue decomposition, and their reported robust capacity to degrade plant polysaccharides (e.g., cellulose, hemicellulose, and lignin) [45] might aid *Elaphodus cephalophus* in deriving energy from high-fiber diets— a potential functional association that requires further experimental confirmation. Thus, the natural environment of *Elaphodus cephalophus* (i.e., Ya’an Baoxing) might profoundly shape the structure and function of its gastrointestinal microbiota, serving as a key driver behind the region-specific distribution and potential functional adaptation of its gut microbes. In ruminants like *Elaphodus cephalophus*, this compartmentalized microbial distribution might reflect a long-term adaptation to a herbivorous diet, and the productivity and health of ruminants are known to rely on a complex gut ecosystem in which microbiota break down cellulose and synthesize essential nutrients [46]. Microbial diversity has been suggested to maximize feed efficiency and maintain intestinal health [46], and the observations from this case report could provide a foundation for future research to explore these potential relationships in *Elaphodus cephalophus*.

## 5. Conclusions

This descriptive case report presents the morphological features of the gastrointestinal muscle layers and gut microbial distribution patterns of a single wild *Elaphodus cephalophus*. The stomach exhibited the thickest circular and longitudinal muscle layers. Bacterial diversity was highest in the stomach and lowest in the ileum, with Firmicutes and Bacteroidota as the dominant phyla. Fungal diversity peaked in the cecum, with Ascomycota and Basidiomycota as the dominant phyla. Each gastrointestinal segment harbored distinct bacterial and fungal genera. These findings provide baseline data for the digestive physiology and gut microbiota of this endangered species, laying a foundation for future comparative studies.

## Figures and Tables

**Table 1:** Key correlation patterns of the gastrointestinal muscle layers in *Elaphodus cephalophus* (Only for this individual study).

Correlation Structure	Correlation Coefficient (*r*)
Gastric ring muscular layer-duodenal ring muscular layer	0.845
Gastric longitudinal muscle layer—duodenal longitudinal muscle layer	0.745

## Data Availability

The datasets generated and analyzed in this study are available in the Supplementary Materials.

## Supplementary Materials

The following supporting information can be downloaded at https://www.mdpi.com/article/10.3390/ani15243651/s1. Figure S1: (A–F) Pie charts showing the relative abundance of dominant bacterial genera in different gastrointestinal tract samples. (A) Rectum, (B) Stomach, (C) Jejunum, (D) Ileum, (E) Duodenum, (F) Cecum.; Figure S2: (A–F) Pie charts of relative abundance at the bacterial genus level in the gastrointestinal tract. (A) Stomach, (B) Duodenum, (C) Jejunum, (D) Ileum, (E) Cecum, (F) Rectum. (G) Bubble plot of bacterial abundance at the phylum level across the gastrointestinal tract. (H) Bubble plot of bacterial abundance at the genus level across the gastrointestinal tract. Table S1: Correlation comparison of the thickness of the mucosal layer, circular muscle layer, and longitudinal muscle layer in the gastrointestinal tract.; Table S2: Thickness of the mucosal layer, circular muscle layer, and longitudinal muscle layer in the gastrointestinal tract (μm).

## Abbreviations

The following abbreviations are used in this manuscript:

GIT	Gastrointestinal Tract
ITS	Internal Transcribed Spacer
OTU	Operational Taxonomic Unit
ASV	Amplicon Sequence Variant
PCoA	Principal Coordinate Analysis
PLS-DA	Partial Least Squares Discriminant Analysis
SCFAs	Short-Chain Fatty Acids
IBD	Inflammatory Bowel Disease
