# Supplementary material for: Gut Morphological Structure-Microbial Characteristics in Elaphodus cephalophus: A Case Report
Source: Animals (Basel). 2025 Dec 18;15(24):3651. doi: 10.3390/ani15243651 (PMC12729593; doi:10.3390/ani15243651)
Supplement: Supplementary file 1 [file animals-15-03651-s001.zip › animals-3971911-supplementary.pdf]

Table S1. Correlation comparison of the thickness of the mucosal layer, circular muscle layer, and longitudinal muscle layer in the gastrointestinal tract. (Only for this individual study)

		Duodenum 50x Mucosal Layer	Duodenum-100x Circular Muscle	Duodenum-100x Longitudinal Muscle	Rectum Mucosal Layer
Duodenum 50x Mucosal Layer	Pearson Correlation	1	.707*	.208	-.771*
	Sig. (2-tailed)		.050	.622	.025
	N	8	8	8	8
Duodenum-100x Circular Muscle	Pearson Correlation	.707*	1	.066	-.744*
	Sig. (2-tailed)	.050		.877	.034
	N	8	8	8	8
Duodenum-100x Longitudinal Muscle	Pearson Correlation	.208	.066	1	-.186
	Sig. (2-tailed)	.622	.877		.660
	N	8	8	8	8
Rectum Mucosal Layer	Pearson Correlation	-.771*	-.744*	-.186	1
	Sig. (2-tailed)	.025	.034	.660	
	N	8	8	8	8
Rectum Circular Muscle	Pearson Correlation	.061	-.501	-.323	.296
	Sig. (2-tailed)	.885	.206	.435	.476
	N	8	8	8	8
Rectum Longitudinal Muscle	Pearson Correlation	-.119	.128	-.039	.406
	Sig. (2-tailed)	.778	.762	.927	.318
	N	8	8	8	8
Rectum Longitudinal Section Mucosal Layer	Pearson Correlation	-.063	.416	-.389	.002
	Sig. (2-tailed)	.882	.305	.341	.997
	N	8	8	8	8
Rectum Longitudinal Section Longitudinal	Pearson Correlation	-.534	-.435	-.127	.504
	Sig. (2-tailed)	.173	.281	.765	.203

Muscle	N	8	8	8	8
Rectum Longitudinal Section Circular Muscle	Pearson Correlation	.025	.154	-.195	.357
	Sig. (2-tailed)	.953	.716	.644	.385
	N	8	8	8	8
Gastric Mucosal Layer	Pearson Correlation	-.781*	-.656	-.412	.620
	Sig. (2-tailed)	.022	.077	.311	.101
	N	8	8	8	8
Gastric Circular Muscle	Pearson Correlation	.616	.840**	.338	-.757*
	Sig. (2-tailed)	.104	.009	.413	.030
	N	8	8	8	8
Gastric Longitudinal Muscle	Pearson Correlation	.333	.135	.746*	-.255
	Sig. (2-tailed)	.421	.751	.034	.541

Continuation Table S1

		Rectum Circular Muscle	Rectum Longitudinal Muscle	Rectum Longitudinal Section Mucosal Layer	Rectum Longitudinal Section Longitudinal Muscle
Duodenum 50x Mucosal Layer	Pearson Correlation	.061	-.119*	-.063	-.534*
	Sig. (2-tailed)	.885	.778	.882	.173
	N	8	8	8	8
Duodenum-100x Circular Muscle	Pearson Correlation	-.501*	.128	.416	-.435*
	Sig. (2-tailed)	.206	.762	.305	.281
	N	8	8	8	8
Duodenum-100x	Pearson Correlation	-.323	-.039	-.389	-.127

Longitudinal Muscle	Sig. (2-tailed)	.435	.927	.341	.765
	N	8	8	8	8
Rectum Mucosal Layer	Pearson Correlation	.296*	.406*	.002	.504
	Sig. (2-tailed)	.476	.318	.997	.203
	N	8	8	8	8
Rectum Circular Muscle	Pearson Correlation	1	.109	-.149	-.130
	Sig. (2-tailed)		.797	.725	.759
	N	8	8	8	8
Rectum Longitudinal Muscle	Pearson Correlation	.109	1	.737	-.232
	Sig. (2-tailed)	.797		.037	.580
	N	8	8	8	8
Gastric Mucosal Layer	Pearson Correlation	.198*	-.113	-.148	.731
	Sig. (2-tailed)	.638	.791	.726	.039
	N	8	8	8	8
Gastric Circular Muscle	Pearson Correlation	-.721	-.290**	-.045	-.156*
	Sig. (2-tailed)	.043	.486	.916	.712
	N	8	8	8	8
Gastric Longitudinal Muscle	Pearson Correlation	-.307	-.170	-.376*	-.413
	Sig. (2-tailed)	.460	.688	.358	.309

Continuation Table S1

		Rectum Longitudinal Section Circular Muscle	Gastric Mucosal Layer	Gastric Circular Muscle	Gastric Longitudinal Muscle	Cecum Longitudinal Muscle
Duodenum 50x Mucosal Layer	Pearson Correlation	.025	-.781*	.616	.333*	-.641
	Sig. (2-tailed)	.953	.022	.104	.421	.087
	N	8	8	8	8	8
Duodenum-100x Circular Muscle	Pearson Correlation	.154*	-.656	.840	.135*	-.295
	Sig. (2-tailed)	.716	.077	.009	.751	.478
	N	8	8	8	8	8
Duodenum-100x Longitudinal Muscle	Pearson Correlation	-.195	-.412	.338	.746	-.566
	Sig. (2-tailed)	.644	.311	.413	.034	.143
	N	8	8	8	8	8
Rectum Mucosal Layer	Pearson Correlation	.357*	.620*	-.757	-.255	.370
	Sig. (2-tailed)	.385	.101	.030	.541	.366
	N	8	8	8	8	8
Rectum Circular Muscle	Pearson Correlation	.218	.198	-.721	-.307	-.237
	Sig. (2-tailed)	.604	.638	.043	.460	.571
	N	8	8	8	8	8
Rectum Longitudinal Muscle	Pearson Correlation	.923	-.113	-.290	-.170	-.122
	Sig. (2-tailed)	.001	.791	.486	.688	.774
	N	8	8	8	8	8
Rectum Longitudinal Section Mucosal Layer	Pearson Correlation	.735	-.148	-.045	-.376	.210
	Sig. (2-tailed)	.038	.726	.916	.358	.618
	N	8	8	8	8	8
Rectum Longitudinal Section Longitudinal	Pearson Correlation	-.179	.731	-.156	-.413	.239
	Sig. (2-tailed)	.671	.039	.712	.309	.569

Muscle	N	8	8	8	8	8
Rectum Longitudinal Section Circular Muscle	Pearson Correlation	1	-.199	-.283	-.210	-.078
	Sig. (2-tailed)		.636	.497	.617	.855
	N	8	8	8	8	8
Gastric Mucosal Layer	Pearson Correlation	-.199*	1	-.566	-.699	.363
	Sig. (2-tailed)	.636		.143	.054	.377
	N	8	8	8	8	8
Gastric Circular Muscle	Pearson Correlation	-.283	-.566**	1	.386*	-.249*
	Sig. (2-tailed)	.497	.143		.345	.551
	N	8	8	8	8	8
Gastric Longitudinal Muscle	Pearson Correlation	-.210	-.699	.386*	1	-.180
	Sig. (2-tailed)	.617	.054	.345		.670

Continuation Table S1

		Cecum Circular Muscle	Cecum Villous Layer	Ileum Longitudinal Muscle	Ileum Circular Muscle
Duodenum 50x Mucosal Layer	Pearson Correlation	.066	-.247*	-.598	.216*
	Sig. (2-tailed)	.877	.556	.117	.608
	N	8	8	8	8
Duodenum-100x Circular Muscle	Pearson Correlation	.127*	-.591	-.297	.347*
	Sig. (2-tailed)	.764	.123	.476	.399
	N	8	8	8	8
Duodenum-100x Longitudinal Muscle	Pearson Correlation	.514	-.029	-.681	.326
	Sig. (2-tailed)	.192	.946	.063	.430
	N	8	8	8	8
Rectum Mucosal Layer	Pearson Correlation	.181*	.581*	.550	-.016
	Sig. (2-tailed)	.669	.131	.158	.970

	N	8	8	8	8
Rectum Circular Muscle	Pearson Correlation	-.010	.603	-.015	-.414
	Sig. (2-tailed)	.981	.113	.972	.307
	N	8	8	8	8
Rectum Longitudinal Muscle	Pearson Correlation	.768	.246	.240	.375
	Sig. (2-tailed)	.026	.557	.566	.361
	N	8	8	8	8
Rectum Longitudinal Section Mucosal Layer	Pearson Correlation	.357	-.126	.464	.293
	Sig. (2-tailed)	.385	.766	.247	.482
	N	8	8	8	8
Rectum Longitudinal Section Longitudinal Muscle	Pearson Correlation	-.515	.510	.301	-.298
	Sig. (2-tailed)	.192	.197	.468	.473
	N	8	8	8	8
Rectum Longitudinal Section Circular Muscle	Pearson Correlation	.590	.364	.374	.459
	Sig. (2-tailed)	.124	.376	.362	.252
	N	8	8	8	8
Gastric Mucosal Layer	Pearson Correlation	-.444*	.430	.360	-.702
	Sig. (2-tailed)	.271	.287	.380	.052
	N	8	8	8	8
Gastric Circular Muscle	Pearson Correlation	-.090	-.639**	-.435	.320*
	Sig. (2-tailed)	.832	.088	.281	.440
	N	8	8	8	8
Gastric Longitudinal Muscle	Pearson Correlation	.428	-.376	-.393*	.648
	Sig. (2-tailed)	.290	.358	.336	.083

Continuation Table S1

		Ileum Villous Layer	Jejunum Villous Layer	Jejunum Circular Muscle	Jejunum Longitudinal Muscle
Duodenum 50x Mucosal Layer	Pearson Correlation	.018	.750*	.476	.816*
	Sig. (2-tailed)	.966	.032	.233	.014
	N	8	8	8	8
Duodenum-100x Circular Muscle	Pearson Correlation	-.076*	.534	-.004	.547*
	Sig. (2-tailed)	.858	.173	.992	.161
	N	8	8	8	8
Duodenum-100x Longitudinal Muscle	Pearson Correlation	.215	.340	.648	-.322
	Sig. (2-tailed)	.609	.410	.082	.437
	N	8	8	8	8
Rectum Mucosal Layer	Pearson Correlation	.508*	-.334*	-.174	-.496
	Sig. (2-tailed)	.198	.418	.681	.211
	N	8	8	8	8
Rectum Circular Muscle	Pearson Correlation	.245	.019	.311	.320
	Sig. (2-tailed)	.559	.964	.454	.440
	N	8	8	8	8
Rectum Longitudinal Muscle	Pearson Correlation	.781	.076	.184	.041
	Sig. (2-tailed)	.022	.859	.663	.924
	N	8	8	8	8
Rectum Longitudinal Section Mucosal Layer	Pearson Correlation	.278	-.224	-.169	.201
	Sig. (2-tailed)	.505	.594	.689	.633
	N	8	8	8	8

Rectum Longitudinal Section Longitudinal Muscle	Pearson Correlation	.037	-.093	-.556	-.462
	Sig. (2-tailed)	.931	.826	.152	.249
	N	8	8	8	8
Rectum Longitudinal Section Circular Muscle	Pearson Correlation	.832	.124	.209	.313
	Sig. (2-tailed)	.010	.770	.620	.451
	N	8	8	8	8
Gastric Mucosal Layer	Pearson Correlation	-.177*	-.444	-.667	-.602
	Sig. (2-tailed)	.674	.270	.071	.115
	N	8	8	8	8
Gastric Circular Muscle	Pearson Correlation	-.285	.533**	-.039	.278*
	Sig. (2-tailed)	.494	.173	.926	.505
	N	8	8	8	8
Gastric Longitudinal Muscle	Pearson Correlation	.080	.219	.670*	-.001
	Sig. (2-tailed)	.851	.603	.069	.998

Continuation Table S1

		Duodenum 50x Mucosal Layer	Duodenum-100x Circular Muscle	Duodenum-100x Longitudinal Muscle	Rectum Mucosal Layer
Gastric Longitudinal Muscle	N	8	8*	8	8*
Cecum Longitudinal Muscle	Pearson Correlation	-.641	-.295	-.566	.370
	Sig. (2-tailed)	.087	.478	.143	.366
	N	8*	8	8	8*
Cecum Circular Muscle	Pearson Correlation	.066	.127	.514	.181
	Sig. (2-tailed)	.877	.764	.192	.669
	N	8	8	8	8

Cecum Villous Layer	Pearson Correlation	-.247	-.591	-.029	.581
	Sig. (2-tailed)	.556	.123	.946	.131
	N	8*	8*	8	8
Ileum Longitudinal Muscle	Pearson Correlation	-.598	-.297	-.681	.550
	Sig. (2-tailed)	.117	.476	.063	.158
	N	8	8	8	8
Ileum Circular Muscle	Pearson Correlation	.216	.347	.326	-.016
	Sig. (2-tailed)	.608	.399	.430	.970
	N	8	8	8	8
Ileum Villous Layer	Pearson Correlation	.018	-.076	.215	.508
	Sig. (2-tailed)	.966	.858	.609	.198
	N	8	8	8	8
Jejunum Villous Layer	Pearson Correlation	.750	.534	.340	-.334
	Sig. (2-tailed)	.032	.173	.410	.418
	N	8	8	8	8
Jejunum Circular Muscle	Pearson Correlation	.476	-.004	.648	-.174
	Sig. (2-tailed)	.233	.992	.082	.681
	N	8	8	8	8
Jejunum Longitudinal Muscle	Pearson Correlation	.816	.547	-.322	-.496
	Sig. (2-tailed)	.014	.161	.437	.211
	N	8*	8	8	8

Continuation Table S1

		Rectum Circular Muscle	Rectum Longitudinal Muscle	Rectum Longitudinal Section Mucosal Layer	Rectum Longitudinal Section Longitudinal Muscle
Gastric Longitudinal Muscle	N	8	8*	8	8*
Cecum Longitudinal Muscle	Pearson Correlation	-.237	-.122	.210	.239
	Sig. (2-tailed)	.571	.774	.618	.569
	N	8*	8	8	8*
Cecum Circular Muscle	Pearson Correlation	-.010	.768	.357	-.515
	Sig. (2-tailed)	.981	.026	.385	.192
	N	8	8	8	8
Cecum Villous Layer	Pearson Correlation	.603	.246	-.126	.510
	Sig. (2-tailed)	.113	.557	.766	.197
	N	8*	8*	8	8
Ileum Longitudinal Muscle	Pearson Correlation	-.015	.240	.464	.301
	Sig. (2-tailed)	.972	.566	.247	.468
	N	8	8	8	8
Ileum Circular Muscle	Pearson Correlation	-.414	.375	.293	-.298
	Sig. (2-tailed)	.307	.361	.482	.473

	N	8	8	8	8
Ileum Villous Layer	Pearson Correlation	.245	.781	.278	.037
	Sig. (2-tailed)	.559	.022	.505	.931
	N	8	8	8	8
Jejunum Villous Layer	Pearson Correlation	.019	.076	-.224	-.093
	Sig. (2-tailed)	.964	.859	.594	.826
	N	8	8	8	8
Jejunum Circular Muscle	Pearson Correlation	.311	.184	-.169	-.556
	Sig. (2-tailed)	.454	.663	.689	.152
	N	8	8	8	8
Jejunum Longitudinal Muscle	Pearson Correlation	.320	.041	.201	-.462
	Sig. (2-tailed)	.440	.924	.633	.249
	N	8*	8	8	8

Continuation Table S1

		Rectum Longitudinal Section Circular Muscle	Gastric Mucosal Layer	Gastric Circular Muscle	Gastric Longitudinal Muscle	Cecum Longitudinal Muscle
Gastric Longitudinal Muscle	N	8	8*	8	8*	8
Cecum Longitudinal Muscle	Pearson Correlation	-.078	.363	-.249	-.180	1
	Sig. (2-tailed)	.855	.377	.551	.670	
	N	8*	8	8	8*	8
Cecum Circular Muscle	Pearson Correlation	.590	-.444	-.090	.428	-.358
	Sig. (2-tailed)	.124	.271	.832	.290	.385

	N	8	8	8	8	8
Cecum Villous Layer	Pearson Correlation	.364	.430	-.639	-.376	-.258
	Sig. (2-tailed)	.376	.287	.088	.358	.537
	N	8*	8*	8	8	8
Ileum Longitudinal Muscle	Pearson Correlation	.374	.360	-.435	-.393	.863
	Sig. (2-tailed)	.362	.380	.281	.336	.006
	N	8	8	8	8	8
Ileum Circular Muscle	Pearson Correlation	.459	-.702	.320	.648	.114
	Sig. (2-tailed)	.252	.052	.440	.083	.789
	N	8	8	8	8	8
Ileum Villous Layer	Pearson Correlation	.832	-.177	-.285	.080	-.317
	Sig. (2-tailed)	.010	.674	.494	.851	.444
	N	8	8	8	8	8
Jejunum Villous Layer	Pearson Correlation	.124	-.444	.533	.219	-.761
	Sig. (2-tailed)	.770	.270	.173	.603	.028
	N	8	8	8	8	8
Jejunum Circular Muscle	Pearson Correlation	.209	-.667	-.039	.670	-.600
	Sig. (2-tailed)	.620	.071	.926	.069	.116
	N	8	8	8	8	8
Jejunum Longitudinal Muscle	Pearson Correlation	.313	-.602	.278	-.001	-.270
	Sig. (2-tailed)	.451	.115	.505	.998	.518
	N	8*	8	8	8	8

Continuation Table S1

		Cecum Circular Muscle	Cecum Villous Layer	Ileum Longitudinal Muscle	Ileum Circular Muscle
Gastric Longitudinal Muscle	N	8	8*	8	8*
Cecum Longitudinal Muscle	Pearson Correlation	-.358	-.258	.863	.114
	Sig. (2-tailed)	.385	.537	.006	.789
	N	8*	8	8	8*
Cecum Circular Muscle	Pearson Correlation	1	.004	-.210	.529
	Sig. (2-tailed)		.992	.618	.177
	N	8	8	8	8
Cecum Villous Layer	Pearson Correlation	.004	1	.116	-.236
	Sig. (2-tailed)	.992		.785	.573
	N	8*	8*	8	8
Ileum Longitudinal Muscle	Pearson Correlation	-.210	.116	1	.203
	Sig. (2-tailed)	.618	.785		.630
	N	8	8	8	8
Ileum Circular Muscle	Pearson Correlation	.529	-.236	.203	1
	Sig. (2-tailed)	.177	.573	.630	
	N	8	8	8	8
Ileum Villous Layer	Pearson Correlation	.668	.588	.103	.493
	Sig. (2-tailed)	.070	.125	.808	.215
	N	8	8	8	8
Jejunum Villous Layer	Pearson Correlation	.188	.041	-.641	.141
	Sig. (2-tailed)	.655	.924	.087	.739
	N	8	8	8	8

Jejunum Circular Muscle	Pearson Correlation	.620	.179	-.501	.414
	Sig. (2-tailed)	.101	.671	.206	.308
	N	8	8	8	8
Jejunum Longitudinal Muscle	Pearson Correlation	-.089	-.086	-.080	.217
	Sig. (2-tailed)	.834	.840	.851	.606
	N	8*	8	8	8

Correlation

		Ileum Villous Layer	Jejunum Villous Layer	Jejunum Circular Muscle	Jejunum Longitudinal Muscle
Gastric Longitudinal Muscle	N	8	8*	8	8*
Cecum Longitudinal Muscle	Pearson Correlation	-.317	-.761	-.600	-.270
	Sig. (2-tailed)	.444	.028	.116	.518
	N	8*	8	8	8*
Cecum Circular Muscle	Pearson Correlation	.668	.188	.620	-.089
	Sig. (2-tailed)	.070	.655	.101	.834
	N	8	8	8	8
Cecum Villous Layer	Pearson Correlation	.588	.041	.179	-.086
	Sig. (2-tailed)	.125	.924	.671	.840
	N	8*	8*	8	8

Ileum Longitudinal Muscle	Pearson Correlation	.103	-.641	-.501	-.080
	Sig. (2-tailed)	.808	.087	.206	.851
	N	8	8	8	8
Ileum Circular Muscle	Pearson Correlation	.493	.141	.414	.217
	Sig. (2-tailed)	.215	.739	.308	.606
	N	8	8	8	8
Ileum Villous Layer	Pearson Correlation	1	.345	.451	.115
	Sig. (2-tailed)		.403	.262	.787
	N	8	8	8	8
Jejunum Villous Layer	Pearson Correlation	.345	1	.314	.499
	Sig. (2-tailed)	.403		.449	.208
	N	8	8	8	8
Jejunum Circular Muscle	Pearson Correlation	.451	.314	1	.257
	Sig. (2-tailed)	.262	.449		.540
	N	8	8	8	8
Jejunum Longitudinal Muscle	Pearson Correlation	.115	.499	.257	1
	Sig. (2-tailed)	.787	.208	.540	
	N	8*	8	8	8

*All Pearson Correlations are for reference only.

Table S2. Thickness of the mucosal layer, circular muscle layer, and longitudinal muscle layer in the gastrointestinal tract (μm).

Duodenum

Replicate	Mucosal Layer	Circular Muscle Layer	Longitudinal Muscle Layer
1	297.891	223.009	72.288
2	302.289	221.349	64.944
3	343.644	218.439	62.017
4	377.389	231.058	78.734
5	359.065	229.232	83.846
6	359.112	231.448	75.227
7	307.199	227.501	63.330
8	288.067	213.029	85.273

Jejunum

Replicate	Mucosal Layer	Circular Muscle Layer	Longitudinal Muscle Layer
1	711.824	416.875	83.959
2	704.118	417.303	87.443
3	705.616	426.978	93.764
4	808.329	424.446	91.330
5	719.484	445.993	89.334
6	728.657	423.005	92.128
7	673.473	398.073	83.668
8	688.510	426.560	76.665

Ileum

Replicate	Mucosal Layer	Circular Muscle Layer	Longitudinal Muscle Layer
1	507.487	210.010	104.000
2	448.524	224.009	110.018
3	397.941	166.012	94.085
4	420.193	180.100	54.000
5	432.136	228.035	68.029
6	388.952	258.070	96.000
7	281.009	154.000	94.085
8	380.767	184.000	78.000

Cecum

Replicate	Mucosal Layer	Circular Muscle Layer	Longitudinal Muscle Layer
1	97.324	88.363	108.000
2	78.102	92.087	120.000
3	94.340	60.166	108.000
4	82.219	78.588	92.195
5	76.236	110.218	98.671
6	73.376	68.469	114.630
7	69.857	58.310	118.423
8	86.833	76.105	110.164

Rectum

Replicate	Mucosal Layer	Circular Muscle Layer	Longitudinal Muscle Layer
1	157.806	206.773	51.158
2	160.853	211.229	49.038
3	144.068	224.146	42.515
4	134.887	209.088	44.022
5	131.943	208.874	48.695
6	131.820	197.876	41.517
7	134.641	202.014	41.908
8	153.851	207.484	40.322

Stomach

Replicate	Mucosal Layer	Circular Muscle Layer	Longitudinal Muscle Layer
1	166.267	468.501	364.067
2	152.112	462.007	386.586
3	154.901	451.533	368.761
4	139.221	514.613	385.691
5	107.446	489.134	405.694
6	113.811	525.107	406.540
7	170.714	495.177	362.366
8	168.989	468.293	401.941

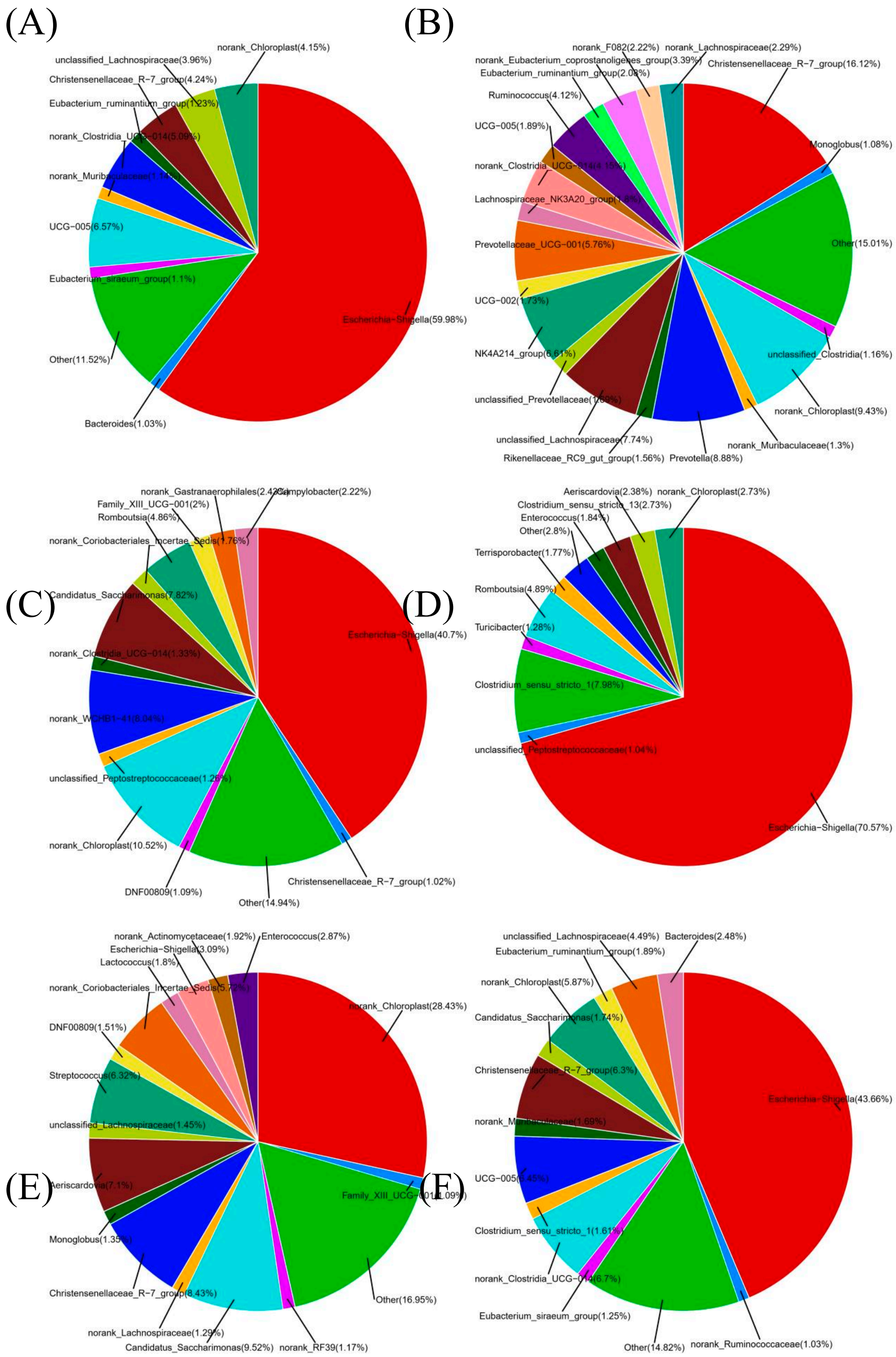

Figure S1. (A-F) Pie charts showing the relative abundance of dominant bacterial genera in different gastrointestinal tract samples. (A) Rectum, (B) Stomach, (C) Jejunum, (D) Ileum,

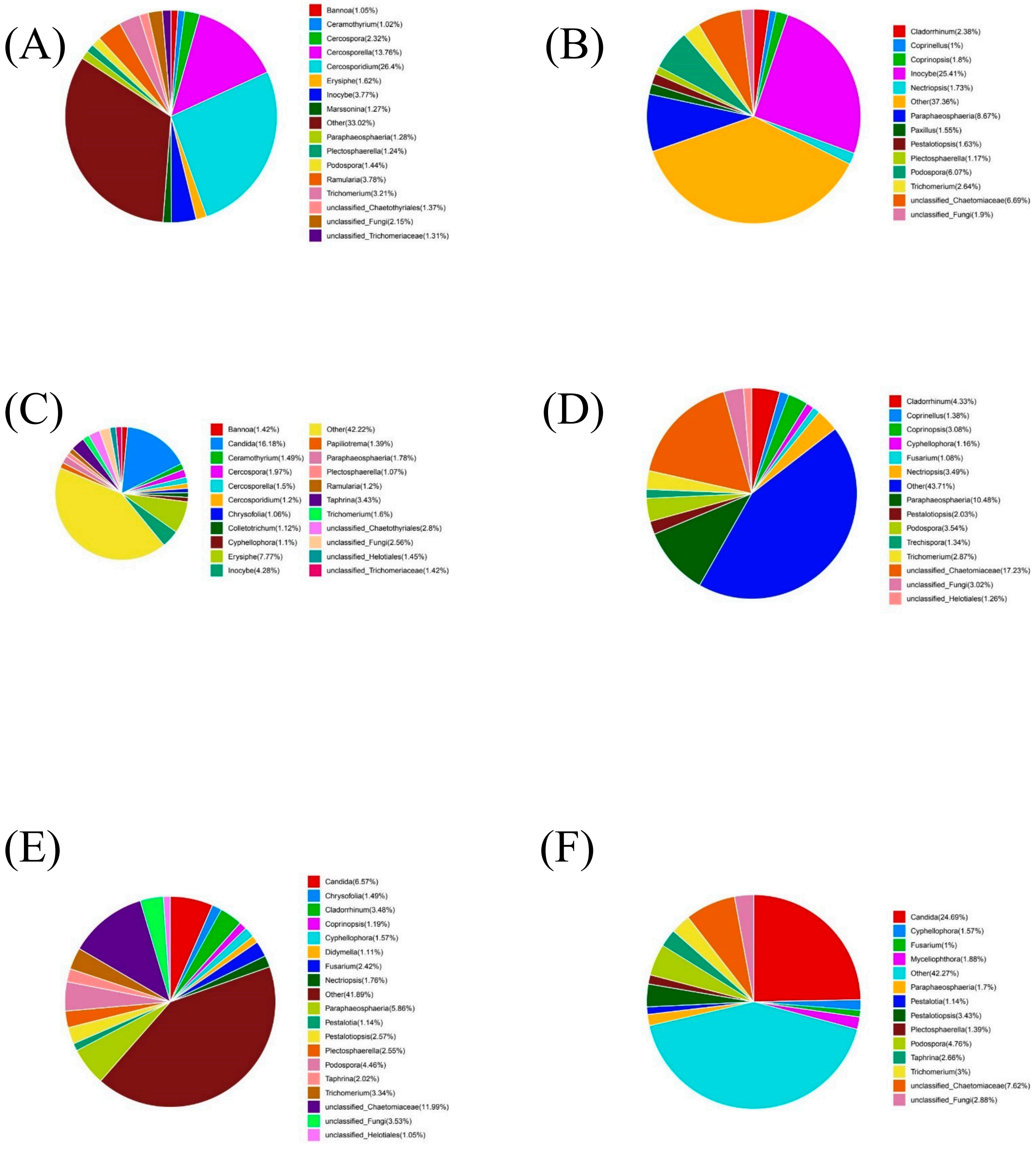
(A)
Bannoa(1.05%)
Ceramothyrium(1.02%)
Cercospora(2.32%)
Cercosporella(13.76%)
Cercosporidium(26.4%)
Erysiphe(1.62%)
Inocybe(3.77%)
Marssonina(1.27%)
Other(33.02%)
Paraphaeosphaeria(1.28%)
Plectosphaerella(1.24%)
Podospora(1.44%)
Ramularia(3.78%)
Trichomerium(3.21%)
unclassified_Chaetothyriales(1.37%)
unclassified_Fungi(2.15%)
unclassified_Trichomeriaceae(1.31%)
(B)
Cladorrhinum(2.38%)
Coprinellus(1%)
Coprinopsis(1.8%)
Inocybe(25.41%)
Nectriopsis(1.73%)
Other(37.36%)
Paraphaeosphaeria(8.67%)
Paxillus(1.55%)
Pestalotiopsis(1.63%)
Plectosphaerella(1.17%)
Podospora(6.07%)
Trichomerium(2.64%)
unclassified_Chaetomiaceae(6.69%)
unclassified_Fungi(1.9%)
(C)
Bannoa(1.42%)
Candida(16.18%)
Ceramothyrium(1.49%)
Cercospora(1.97%)
Cercosporella(1.5%)
Cercosporidium(1.2%)
Chrysofolia(1.06%)
Colletotrichum(1.12%)
Cyphellophora(1.1%)
Erysiphe(7.77%)
Inocybe(4.28%)
Other(42.22%)
Papiliotrema(1.39%)
Paraphaeosphaeria(1.78%)
Plectosphaerella(1.07%)
Ramularia(1.2%)
Taphrina(3.43%)
Trichomerium(1.6%)
unclassified_Chaetothyriales(2.8%)
unclassified_Fungi(2.56%)
unclassified_Helotiales(1.45%)
unclassified_Trichomeriaceae(1.42%)
(D)
Cladorrhinum(4.33%)
Coprinellus(1.38%)
Coprinopsis(3.08%)
Cyphellophora(1.16%)
Fusarium(1.08%)
Nectriopsis(3.49%)
Other(43.71%)
Paraphaeosphaeria(10.48%)
Pestalotiopsis(2.03%)
Podospora(3.54%)
Trechispora(1.34%)
Trichomerium(2.87%)
unclassified_Chaetomiaceae(17.23%)
unclassified_Fungi(3.02%)
unclassified_Helotiales(1.26%)
(E)
Candida(6.57%)
Chrysofolia(1.49%)
Cladorrhinum(3.48%)
Coprinopsis(1.19%)
Cyphellophora(1.57%)
Didymella(1.11%)
Fusarium(2.42%)
Nectriopsis(1.76%)
Other(41.89%)
Paraphaeosphaeria(5.86%)
Pestalotia(1.14%)
Pestalotiopsis(2.57%)
Plectosphaerella(2.55%)
Podospora(4.46%)
Taphrina(2.02%)
Trichomerium(3.34%)
unclassified_Chaetomiaceae(11.99%)
unclassified_Fungi(3.53%)
unclassified_Helotiales(1.05%)
(F)
Candida(24.69%)
Cyphellophora(1.57%)
Fusarium(1%)
Myceliophthora(1.88%)
Other(42.27%)
Paraphaeosphaeria(1.7%)
Pestalotia(1.14%)
Pestalotiopsis(3.43%)
Plectosphaerella(1.39%)
Podospora(4.76%)
Taphrina(2.66%)
Trichomerium(3%)
unclassified_Chaetomiaceae(7.62%)
unclassified_Fungi(2.88%)

(G)

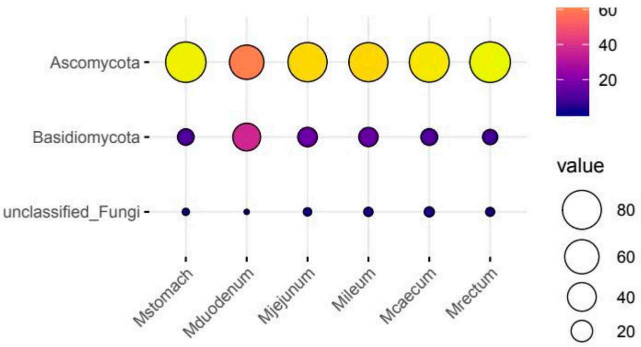

(H)

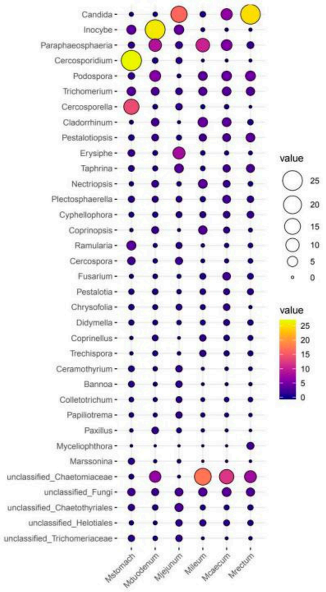

Figure S2. (A-F) Pie charts of relative abundance at the fungal genus level in the gastrointestinal tract. (A) Stomach, (B) Duodenum, (C) Jejunum, (D) Ileum, (E) Cecum, (F) Rectum. (G) Bubble plot of fungal abundance at the phylum level across the gastrointestinal tract. (H) Bubble plot of fungal abundance at the genus level across the gastrointestinal tract.
